# Thinner temporal peripapillary retinal nerve fibre layer in Stargardt disease detected by optical coherence tomography

**DOI:** 10.1007/s00417-020-04992-2

**Published:** 2020-11-13

**Authors:** Michael Reich, Jan Lübke, Lutz Joachimsen, Julia Stifter, Sebastian Küchlin, Daniel Böhringer, Clemens Lange, Wolf A. Lagrèze

**Affiliations:** Eye Center, Medical Center - University of Freiburg, Faculty of Medicine, University of Freiburg, Killianstrasse 5, D-79106 Freiburg, Germany

**Keywords:** Anterograde transneuronal degeneration, ABCA4, Stargardt disease, Retinal nerve fibre layer, Optic disc

## Abstract

**Purpose:**

To evaluate peripapillary retinal nerve fibre layer (RNFL) thickness measured by spectral domain optical coherence tomography (OCT) in patients with Stargardt disease (STGD).

**Methods:**

A cross-sectional, monocentric, observational case-control study. Twenty patients (39 eyes) with ABCA4 mutations graded according to the Fishman STGD classification were included. RNFL measurement was performed using Heidelberg Spectralis SD-OCT. RNFL thickness in STGD patients was compared to age-matched data of healthy individuals provided by the device’s manufacturer. A manual readjustment of the optic disc-fovea angle was performed when needed.

**Results:**

The mean age at first diagnosis of STGD was 22.9 years (range 9 to 50) and 39.1 years (range 18 to 74) at the time of examination. Thirty-nine percent of eyes (15 eyes) needed manual adjustment of the optic disc-fovea angle due to malfixation of the patients during OCT. The temporal quadrant corresponding to the macula showed a RNFL 16% thinner than controls (mean − 12 μm, 95%CI − 9 to −15 μm). However, global RNFL thickness did not differ from controls due to increased RNFL thickness of 12% in the nasal sectors. Duration and stage of STGD were not correlated to thinner RNFL.

**Conclusion:**

STGD seems to be associated with thinner peripapillary RNFL in the sector of axons projecting to the degenerated macular area. It is yet unclear as to whether this results from anterograde transneuronal degeneration of direct injury to retinal ganglion cells.

**Supplementary Information:**

The online version contains supplementary material available at 10.1007/s00417-020-04992-2.

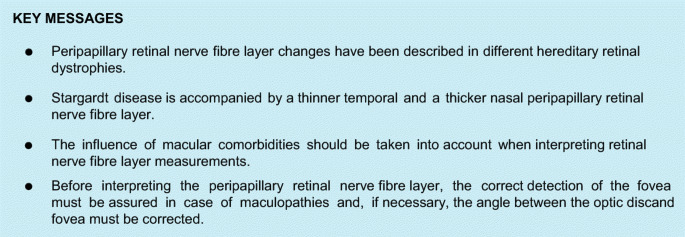

## Introduction

Axonal loss in the peripapillary retinal nerve fibre layer (RNFL) occurs in visual pathway degeneration such as glaucoma, but also ischemic, inflammatory, or compressive optic neuropathies [1–3]. Especially in glaucoma, RNFL changes are an important parameter for estimation of disease progression [1]. In the age group typically affected by glaucoma, macular comorbidities such as age-related macular degeneration (AMD) are highly prevalent. Hence, it is important to know whether and to which extent macular diseases lead to peripapillary RNFL loss potentially interfering with the RNFL analysis. Despite the high prevalence of both AMD and glaucoma, only few studies have addressed this question [4–6], some of them with regard to the question as to whether anti-VEGF therapy interferes with the peripapillary RNFL [7].

To quantify RNFL changes resulting from maculopathies independent of potentially coexisting optic nerve diseases, it may be reasonable to focus on hereditary maculopathies, which typically manifest in younger individuals. The most common one is Stargardt disease (STGD) with a prevalence of about 1:10,000 [8] leading to irreversible bilateral loss of central vision [9]. Fundus examination reveals macular degeneration with a beaten-bronze or bull’s eye appearance and characteristic deep yellowish-white fish-shaped flecks in the macular and perimacular region. The end-stage fundus aspect is characterized by extensive macular atrophy, with resorbed flecks and sparse pigmentation [10]. The underlying pathophysiology of STGD is not yet fully understood. It is generally accepted that mutations in the ABCA4 gene cause ATP-binding cassette (ABC) protein dysfunction, leading to deposition of lipofuscin in RPE cells and consequently malfunction and later atrophy of photoreceptor cells [11].

As a consequence of photoreceptor cell loss, a thinner RNFL has been described in hereditary retinal dystrophies affecting the entire retina [12–14], possibly due to anterograde transsynaptic axonal degeneration. This degeneration is described as “dying-forward” of the downstream neuron caused by damage of the preceding neuron [15–18]. It is assumed to be caused by defective afferent innervation [19] and reduced transmission of growth and survival factors from the supplying cell [15, 16].

In STGD, RNFL changes have so far been analyzed in one study by Genead et al. [20], describing a 46.2% thinner RNFL in 52 eyes, mainly in the superior and inferior quadrants. These results are remarkable since retinal dystrophy in STGD primarily affects the macula, which corresponds to the temporal, papillomacular nerve fibre bundle. Accordingly, a RNFL thinning would be more likely to first occur in the temporal quadrant. Therefore, we analyzed the RNFL measured by spectral domain optical coherence tomography (OCT), to either support or disregard RNFL thinning in association with STGD.

## Methods

### Study design

This cross-sectional, monocentric, observational case-control study was conducted on 20 STDG patients. All patients consulted our eye centre for the first time between 2000 and 2019 and were examined in the study between June 2017 and January 2019. Informed written consent was obtained from all patients. The study was approved by our institutional Ethics Committee and adhered to the tenets of the Declaration of Helsinki. Optical coherence tomography angiography data of some patients were previously been characterized and published [21].

### Inclusion and exclusion criteria

Patients of all STGD stages with detected ABCA4 mutation were included. The stage of disease was graded according to the Fishman STGD classification [22]. Stage (S) 1 is characterized by macular pigmentary changes and irregular pigmentary spots (flecks) located within one disc diameter of the fovea. S2 is identified when the pigmentary changes and pisciform flecks are located beyond the vascular arcades temporally and often extend nasally to the optic disc. S3 is defined by the resorption of previously diagnosed flecks resulting in focal choriocapillary (CC) and retinal pigment epithelium (RPE) atrophy of the macula. S4 is characterized by diffusely absorbed flecks and extensive CC and RPE atrophy throughout the entire central fundus. No patient had any ocular disease other than STGD, especially no glaucoma, or history of ocular hypertension, a refractive error exceeding + 5 or − 7 dioptres (D), or revealed evidence of acquired or hereditary systemic disease according to the medical history of the patients, the eye examination as part of this study, and reports from the referring physicians. Furthermore, patients with congenital optic nerve anomalies were excluded, as well as eyes with poor OCT image quality for example due to moving artefacts.

### Ophthalmologic assessment

**Fig. 1:**
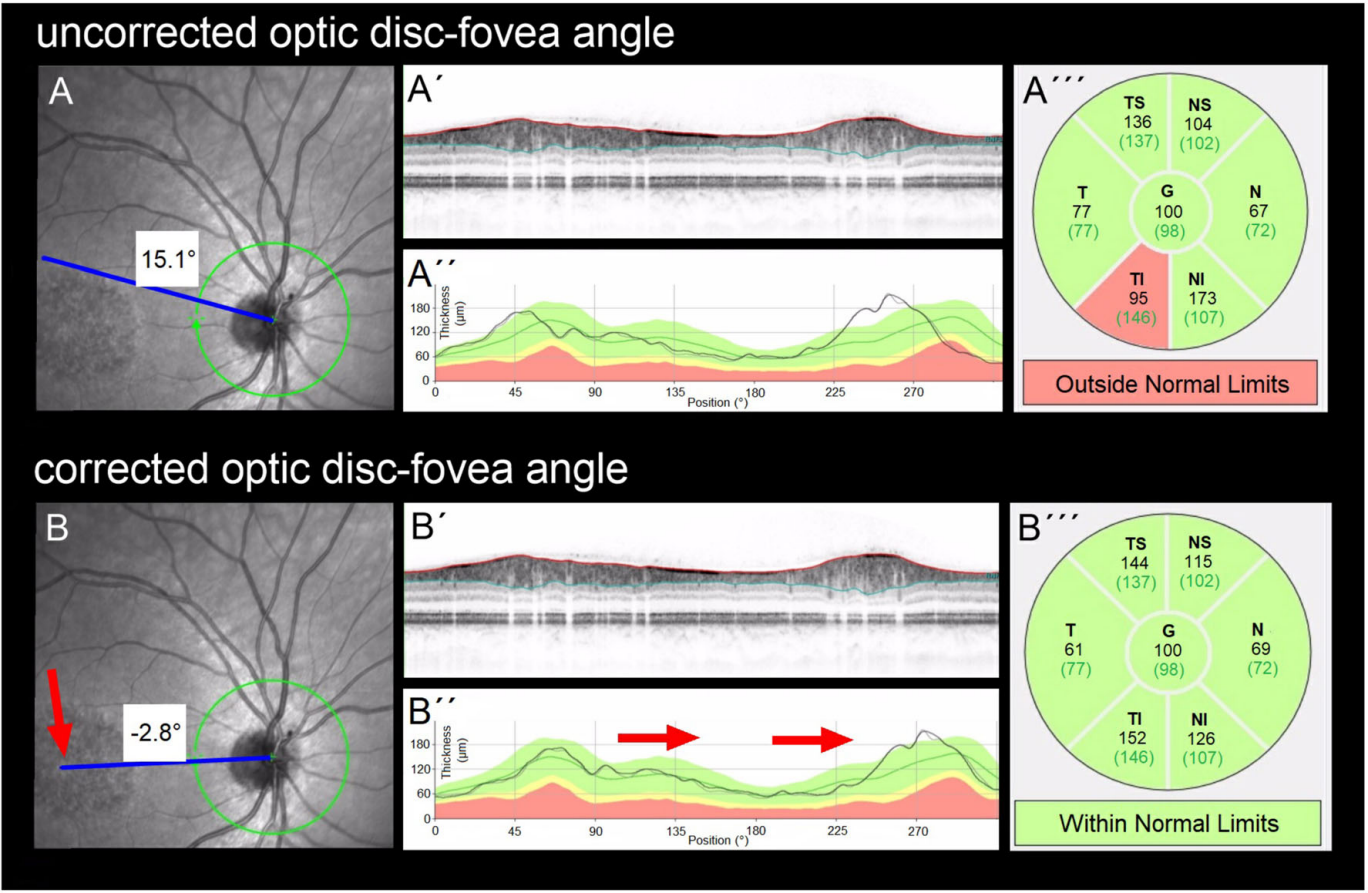
Incorrect peripapillary RNFL thickness measurement due to misalignment to the fovea. Data of an exemplary patient (ID4, see Table 1) are shown. (A) Incorrect measurement results of the peripapillary RNFL thickness due to an incorrect detection of the fovea, resulting in an incorrect thin peripapillary RNFL outside normal limits in the temporal-inferior area (A‴). (B) After manual correction of the optic disc-fovea angle (red arrow, B), the peripapillary RNFL thickness measurement was rotated by a corresponding number of degrees compared to the reference database (in the direction of the red arrows, B″), which resulted in measured normal RNFL thickness in the temporal-inferior area (B‴) and a measured thinner RNFL thickness of the temporal quadrant from 77 μm (A‴) to 61 μm (B‴). The age-correlated 50% percentiles of each sector, which are calculated individually for each patient by the manufacturer software using the normative database described in “Methods,” are illustrated in green numbers

Patients underwent a comprehensive ophthalmological examination including measurement of best-corrected visual acuity (BCVA), assessment of intraocular pressure via Goldmann applanation tonometry, fundus examination, fundus autofluorescence imaging, and macular SD-OCT (version 1.5.12.0, Heidelberg Spectralis, Heidelberg Engineering, Inc., Germany). It has a 3.9-μm axial resolution and scans at 40,000 A-scans per second. For the peripapillary RNFL measurements, the RNFL exam protocol was used for scan acquisition, which was measured automatically by the existing software at a diameter of 3.45 mm around the centre of the optic disc after dilation of the pupil with 1% tropicamide. OCT analysis was performed in accordance with the APOSTEL recommendations for reporting quantitative optical coherence tomography studies [23]. All measurements were performed between 8 and 11 a.m. The same device was used for each patient. Since only high-quality pictures were included, there was no flawed segmentation of the peripapillary RNFL thickness measurements and therefore manual correction was not necessary. The global (G), temporal-inferior (TI, 270 to 315°), temporal (T, 315 to 45°), temporal-superior (TS, 45 to 90°), nasal-superior (NS, 90 to 135°), nasal (N, 135 to 225°), and nasal-inferior (NI, 225 to 270°) RNFL thickness measurements were recorded separately for each sector in micrometres (μm; Fig. 1). The pie charts in Fig. 1 are colour-coded according to percentiles as defined by the manufacturer leading to the following sector classifications: A green sector represents the area above the 5th percentile of the RNFL thickness distribution of eyes in the reference database (within normal limits). A yellow sector represents the area below the 5th percentile, but above the 1st percentile (borderline). A red sector represents the area below the 1st percentile (outside normal limits). The normative data provided by the manufacturer of the OCT device includes 201 healthy Caucasian patients (90/111 females/males) with a mean age of 48.2 ± 14.5 years (range 18 to 78 years) and a refractive error not exceeding + 5 or − 7 D sphere. Peripapillary RNFL thickness decreases slightly with age in healthy patients [24]. The reference database is therefore adapted to the age in order to take this tendency into account. In addition to the colour-coding, the pie charts illustrate the measured RNFL thickness of each sector (black numbers) compared to the age-correlated 50% percentile (green numbers). This age-correlated 50% percentile is calculated individually for each patient by the manufacturer software using the normative database described above. In Fig. 1B‴, for example, the measured RNFL thickness of 61 μm in the temporal sector (black number) is 16 μm thinner—defined in the following as “difference of peripapillary RNFL thickness”—than the age-correlated 50% percentile (77 μm, green number).

If the fovea was not detected correctly by the Heidelberg Eye explorer software, the optic disc-fovea angle (ODFA) was manually corrected as described in Fig. 1 by the grader MR.

### Verification of the manual correction of the optic disc-fovea angle

To verify the correction of the ODFA, an additional grader (SK) performed the manual correction. Differences in peripapillary RNFL thickness compared to age-corrected, normative data provided by the device’s manufacturer of each sector were compared between both graders (MR, SK).

### Statistical analysis

GraphPad PRISM and R were used for statistical analysis. For descriptive data analysis, the mean, standard deviation (SD), and 95% confidence interval (95%CI) were calculated. To evaluate correlation between stage of disease and disease duration, Spearman’s correlation coefficient (rho) was analyzed. For verification of the manual correction of the ODFA, the intraclass correlation coefficient (ICC) was analyzed. A linear mixed model (R package lmerTest) was used to evaluate the influence of the disease stage and duration on the peripapillary RNFL thickness measurements, considering the right-left eye association by including the patient ID as a random factor and including the Fishman STGD stage as a discrete factor level, as well as the disease duration as a linear metric variable. We selected disease duration and Fishman STGD stage as the only variables because we did not record other factors that would make sense including into the model. For example, since it is obvious that the disease duration is correlated with patient age, we did not include the age of the patient in the model. Bonferroni’s correction was used due to multiple testing.

## Results

### Patient characteristics

A total of 39 eyes of 20 STGD patients (12/8 females/males) were included. One eye was excluded due to poor OCT image quality. The average age at initial diagnosis of the disease was 22.9 years (range 9–50) and 39.1 years (range 18–74) at the time of the study examination. Table 1 presents detailed information of the study group. Sixteen eyes of eight patients were assigned to Fishman STGD classification S1, six eyes of three patients to S2, 15 eyes of eight patients to S3, and two eyes of one patient to S4. The stage of disease and the duration of disease was correlated (rho = 0.53, *p* = 0.0006). The range of IOP for all eyes at the time of the study examination was 10 to 20 mmHg (mean 14.7 mmHg).

**Table 1:** Clinical and molecular data of the STGD patients in the study group

ID	Age (ys)	Onset (ys)	Sex	BCVA OD Snellen	BCVA OS Snellen	Fishman STGD classification	Mutation 1	Mutation 2	Mutation 3
1*	40	10	F	20/2000	20/2000	1	c.5196+2T>C (Splice)	c.5882G>A (p.Gly1961Glu)	
2	23	17	F	20/400	20/200	1	c.1622T>C (p.Leu541Pro)	c.3113C>T (p.Ala1038Val)	c.5882G>A (p.Gly1961Glu)
3	27	23	M	20/200	20/200	1	c.4462T>C (p.Cys1488Arg)	c.5882G>A (p.Gly1961Glu)	
4	28	24	F	20/200	20/200	1	c.2588G>C (p.Gly863Ala, Splice)	c.2828G>A (p.Arg943Gln)	c.5603A>T (p.Asn1868Ile)
5	37	36	F	20/40	20/25	1	c.3322C>T (p.Arg1108Cys)	c.5882G>A (pGly1961Glu)	
6	52	42	F	20/200	20/200	1	c.2894A>G (p.Asn965Ser)	c.5882G>A (p.Gly1961Glu)	
7	22	13	M	20/200	20/200	1	c.3322C>T (p.Arg1108Cys)	c.5882G>A (p.Gly1961Ala)	
8	25	14	M	20/200	20/200	1	c.5882G>A (p.Gly1961Glu)	c.6238_6239delTC (p.Ser2080HisfsX16)	
9^#^	18	9	M	20/400	20/200	2	c.2588G>C (p.Gly863Ala, Splice)	c.6238_6239delTC (p.Ser2080HisfsX16)	
10^#^	23	14	F	20/400	20/400	2	c.2588G>C (p.Gly863Ala, Splice)	c.6238_6239delTC (p.Ser2080HisfsX16)	
11	23	21	M	20/80	20/80	2	c.2588G>C (p.Gly863Ala, Splice)	c.858+2T>A (unknown)	
12*	51	22	M	20/320	20/250	3	c.5196+2T>C (Splice)	c.5882G>A (p.Gly1961Glu)	
13	40	23	F	20/160	20/160	3	c.5413A>G (p.Asn1805Asp)	c.5714+5G>A (unknown)	
14*	57	27	F	20/160	20/125	3	c.5196+2T>C (Splice)	c.5882G>A (p.Gly1961Glu)	
15	74	27	M	20/400	20/400	3	c.5882G>A (p.Gly1961Glu)	Deletion exon 18–19	
16	30	27	M	20/400	20/400	3	c.1622T>C (p.Leu541Pro)	c.3113C>T (p.Ala1038Val)	
17	52	25	F	20/400	20/400	3	c.2692G>T (p.Glu898Xaa)	c.5461-10T>C (unknown)	c.5606C>T (p.Pro1869Leu)
18	59	50	F	20/16	20/25	3	c.4610C>T (unknown)	c.5461-10T>C (unknown)	
19	49	20	F	20/400	20/200	3	c.4577C>T (p.Thr1526Met)	c.6415C>T (p.Arg2139Trp)	
20	52	14	F	cf	cf	4	c.2932G>A (p.Gly978Ser)	c.5714+5G>A (unknown)	

*^#^Siblings; *STGD*, Stargardt disease; *ys*, years; *F/M*, female/male; *BCVA*, best-corrected visual acuity; *cf*, counting finger

### Correction of the optic disc-fovea angle

Automatic software detection of the fovea failed in 15 eyes (38.5%) of 11 patients (55%). In these eyes the ODFA was corrected manually. The mean corrected angle between the fovea and the optic nerve centre relative to the horizontal axis defined by the fundus image, was − 4.4 ± 4.7° (range − 16.3 to 0).

### Verification of the manual correction of the optic disc-fovea angle

ICC between differences in peripapillary RNFL thickness compared to the reference database, measured after correction of the ODFA by MR and SK, was high for each sector in the 15 eyes in which the automatic software detection of the fovea failed. TI: ICC 0.978 (CI 0.936 to 0.992, *p* = 4.44E−11); T: ICC 0.998 (CI 0.994 to 0.999, *p* = 4.75E−18); TS: ICC 0.991 (CI 0.972 to 0.997, *p* = 4.88E−14); NS: ICC 0.997 (CI 0.991 to 0.999, *p* = 6.84E−17); N: ICC 0.995 (CI 0.987 to 0.998, *p* = 5.60E−16); NI: ICC 0.991 (CI 0.973 to 0.997, *p* = 1.16E−13). The total volume (G) does not change due to a different correction of the ODFA. Therefore, ICC for G is not given.

### Peripapillary RNFL pattern in Stargardt disease

**Fig. 2:**
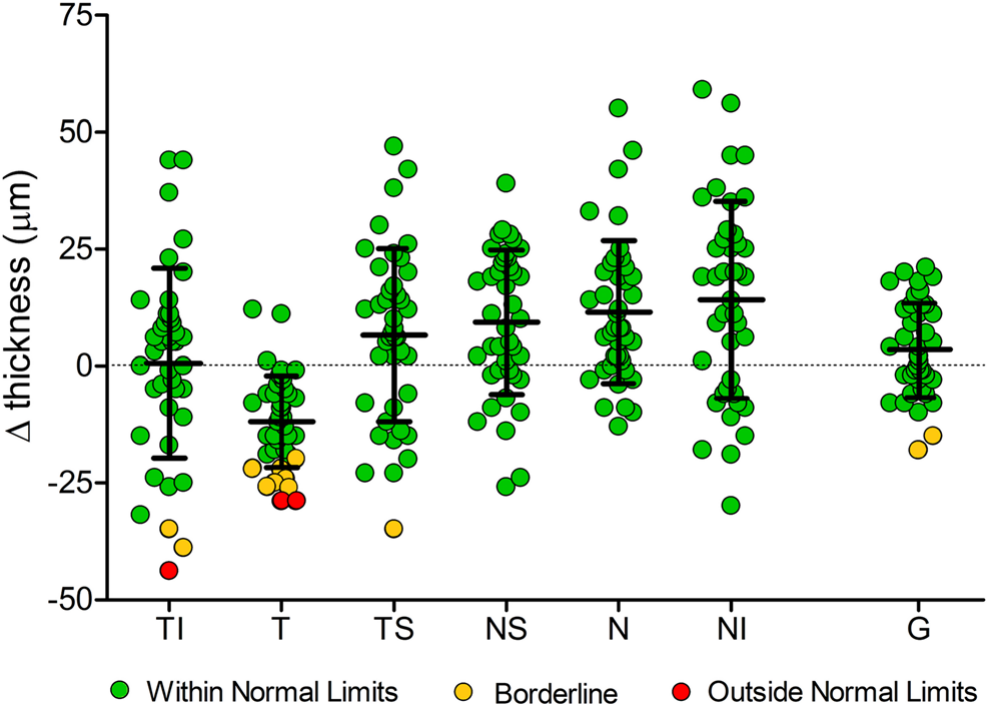
Peripapillary RNFL thickness in Stargardt disease. Differences in peripapillary RNFL thickness (Δ thickness) compared to age-corrected, normative data provided by the manufacturer (Heidelberg Engineering) of each sector are illustrated. “Within normal limits” (green plots) is defined as the area above the 5th percentile of the RNFL thickness distribution in eyes in the reference database, “borderline” (yellow plots) as the area below the 5th percentile, but above the 1st percentile, and “outside normal limits” (red plots) as the area below the 1st percentile. Mean and standard deviation are shown

Differences of peripapillary RNFL thickness in the different sectors compared to the reference database are illustrated in Fig. 2. In the temporal quadrant, 36 of 39 eyes showed thinner peripapillary RNFL (T: mean − 12 μm, 95%CI − 9 to − 15 μm, 16% thinner compared to healthy cohort). Seven of these eyes showed borderline thinner RNFL, and two eyes a thinner RNFL outside normal limits. Since the nasal areas showed peripapillary RNFL thickening (NS: mean 9 μm, 95%CI 4 to 14 μm; N: mean 12 μm, 95%CI 7 to 17 μm; NI: mean 13 μm, 95%CI 6 to 20 μm; in total 12% thickening compared to healthy cohort), the global peripapillary RNFL thickness did not differ from the reference database (G: mean 3 μm, 95%CI 0 to 7 μm), neither did the temporal superior (mean 7 μm, 95%CI 0 to 13 μm) nor the temporal inferior sector (mean 0 μm, 95%CI − 7 to 7 μm). Detailed data on mean thickness and mean thickness difference compared to the age-matched reference database are listed in Table S1. Figure S1 shows that the data in the right eyes are similar to the data in the left eyes. The data divided into the four STGD stages are shown in Fig. S2. Additionally, data of all eyes (*n* = 39) before correction of the ODFA and data of only the 15 eyes needing correction of the ODFA are illustrated in Fig. S3A and Fig. S3B, respectively.

### Correlation between RNFL changes and disease duration and stage

The differences in peripapillary RNFL thickness compared to age-corrected, normative data provided by the device’s manufacturer showed no obvious correlation with the duration of disease and Fishman STGD stage of disease in all RNFL sectors (linear mixed model was used, data not shown).

## Discussion

Analysis of the peripapillary RNFL has become an indispensable diagnostic tool in the clinical assessment of optic nerve diseases. Hence, it is important to know whether macular comorbidities can be associated with additional thinner RNFL, as a potential confounder of progression of the optic neuropathy. This issue has so far only been addressed in few studies which have focused mostly on patients with AMD [4–6]. Due to the older age of patients with AMD, RNFL measurements might be influenced by optic nerve comorbidities. Therefore, hereditary retinal dystrophies appear to be more suitable to analyze the influence of macular atrophy on the RNFL measurement.

Thinner peripapillary RNFL has already been described in different hereditary retinal dystrophies like retinitis pigmentosa [12], autosomal recessive cone-rod dystrophy [13], and X-linked retinoschisis [14] affecting 41.7 to 79% of the patients in at least one eye. So far, only one study by Genead et al. has described a thinner peripapillary RNFL in STGD [20]. In this report, ABCA4 mutations were detected in 19 of the 27 included patients. Thinner peripapillary RNFL was detected in 24 eyes (46.2%) of 14 patients (51.9%) in one or more quadrants. Of these, 33.3% showed thinner RNFL in the superior, 33.3% in the inferior, 16.7% in the nasal, and 16.7% in the temporal quadrant. This distribution is remarkable since retinal dystrophy in STGD primarily affects the macula, corresponding to the temporal optic disc sector, and only reaches beyond this area in advanced stages of disease [22].

Due to macular atrophy, in our study, the fovea could not be automatically detected correctly in 15 eyes (38.5%) of 11 patients (55%). Before correction of the ODFA, we detected thinner RNFL in 17 eyes (43.6%) of 11 patients (55%) in one or more quadrants (54% in the inferior, 33.5% in the temporal, and 12.5% in the superior quadrant; Fig. S3). These data are in accordance with the results published by Genead et al. [20]. Since the authors did not describe a correction of misalignments of RNFL measurements with regard to the fovea, it is possible that the observed pattern of thinner RNFL mainly detected in the inferior quadrant was influenced by a misalignment of the RNFL sectors with regard to the fovea.

Since the correct alignment of the ODFA has no influence on the global RNFL, a thinner global RNFL was detected neither by Genead et al. [20] nor by us. Due to the partly pronounced central retinal atrophy, which renders determination of the fovea difficult, an incorrect alignment of the manual-corrected ODFA cannot be excluded in our study as well. However, this appears unlikely since the ICC of the two graders (MR, SK) was high and the mean corrected ODFA of − 4.4 ± 4.7° in our study is similar to the mean angle of − 7° described by Chauhan and Burgoyne in 222 patients with ocular hypertension or glaucoma [25] and − 7.76° described by Jonas et al. in 3052 individuals older than 50 years independent of ophthalmologic diseases [26].

Anterograde transneuronal degeneration is relatively rare, as neurons usually do not receive just one afferent input. Therefore, the almost one-to-one projection of the foveal neurons [27] makes macular diseases suitable for further analysis. Anterograde transneuronal degeneration has been described in the auditory and the visual systems [17], especially in animal studies [28, 29]. In humans, it has been proposed that retinal ganglion cell damage in glaucoma might induce secondary degeneration of the optic radiation as demonstrated by diffusion-tensor magnetic resonance imaging [30].

We detected an increased RNFL thickness in the nasal sector, where the photoreceptor cells are still functional. The RPE has an important role in ganglion cell neurogenesis [31]. Therefore, one besides many [32] possible explanations for increased RNFL thickness in the nasal sectors is that impaired signalling early in development due to impaired RPE function could result in a more prolonged period of ganglion cell neurogenesis [32]. The highly significant thinner RNFL we detected in the temporal area might be indicative of anterograde transneuronal degeneration due to STGD stage–dependent complete atrophy of macular RPE cells and consecutive degeneration of photoreceptor cells [11]. Nevertheless, due to retinal remodelling processes associated with outer retinal degeneration [33], it might be possible that the detected thinner temporal RNFL is independent of macular atrophy.

A limitation of our study is the lack of a self-generated control group, since only normative data provided by the manufacturer of the OCT device were used for comparison. To further judge on the validity of such a comparison, we reference a study by Bendschneider et al. who determined age-dependent RNFL values in 170 healthy Caucasians using an identical OCT device [34]. Overall, the mean RNFL thickness in the temporal sector was 68.8 μm. Thirty percent of their cohort were younger than 40 years, thus being older than our STGD patients (mean age 39.1 years). Based on their linear correlation, temporal RNFL thickness can be estimated as 72 μm and nasal RNFL thickness as 77 μm at age 39. Compared to these standard values, our STGD patients show a 13% thinner RNFL in the temporal sector and a 10% thicker RNFL in the nasal sector, differing not too much from our results (16% thinner RNFL in the temporal sector, 12% thicker RNFL in the nasal sector). Our study has further certain limitations such as the absence of longitudinal follow-up and the natural of lack of precise retinal ganglion cell density data. Due to extensive macular atrophy, retinal layers could not be segmented in a meaningful way and quantification of the retinal ganglion cell volume via OCT was not possible. Since STGD primarily affects the macula, temporal atrophy of the RNFL seems plausible. In advanced stages, the atrophy extends beyond the macula, so an extension of RNFL loss in advanced stages would also be expected. However, such an advanced loss of the RNFL in advanced STGD stages is not reflected in our data being shown with a linear mixed model, considering the right-left eye association by including patient ID as a random factor and including the Fishman STGD stage as a discrete factor level, as well as the disease duration as a linear metric variable. This might be explained by the small cohort size. In light of these limitations, further prospective, multicentre studies, thus being able to reach a larger cohort size, as well as longitudinal follow-up and ganglion cell density data are warranted to determine the exact extent of RNFL changes in STGD.

In summary, STGD seems to be associated with thinner peripapillary RNFL in the sector of axons projecting to the degenerated macular area. It is yet unclear as to whether this results from anterograde transneuronal degeneration of direct injury to retinal ganglion cells. Due to retinal remodelling processes associated with outer retinal degeneration, it might be possible that the detected temporal thinner peripapillary RNFL is independent of macular atrophy. Nevertheless, the influence of macular comorbidities should be taken into account when interpreting RNFL measurements.

## Supplementary Information

Figure S1 Peripapillary RNFL thickness in Stargardt disease separated between the right and left eye. Differences in peripapillary RNFL thickness (Δ thickness) compared to age-corrected, normative data provided by the manufacturer (Heidelberg Engineering) of each sector are illustrated separately for the right and the left eye. (PNG 61788 kb)

High resolution image (TIFF 1287 kb)

Figure S2 Peripapillary RNFL thickness in different stages of Stargardt disease. The four STGD stages according to the Fishman’s STGD classification are shown (A-D). Differences in peripapillary RNFL thickness (Δ thickness) compared to age-corrected, normative data provided by the manufacturer (Heidelberg Engineering) of each sector are illustrated. “Within normal limits” (green plots) is defined as the area above the 5th percentile of the RNFL thickness distribution in eyes in the reference database, “borderline” (yellow plots) as the area below the 5th percentile, but above the 1st percentile and “outside normal limits” (red plots) as the area below the 1st percentile. Mean and standard deviation are shown. (PNG 3615 kb)

High resolution image (TIF 1621 kb)

Figure S3 Peripapillary RNFL thickness in different stages of Stargardt disease before correction of the optic disc-fovea angle. Data of all 39 eyes before correction of the optic dis-fovea angle (A) and only that 15 eye with misalignment of the optic dis-fovea angle (B) are illustrated. Differences in peripapillary RNFL thickness (Δ thickness) compared to age-corrected, normative data provided by the manufacturer (Heidelberg Engineering) of each sector are illustrated. “Within normal limits” (green plots) is defined as the area above the 5th percentile of the RNFL thickness distribution in eyes in the reference database, “borderline” (yellow plots) as the area below the 5th percentile, but above the 1st percentile and “outside normal limits” (red plots) as the area below the 1st percentile. Mean and standard deviation are shown. (PNG 4557 kb)

High resolution image (TIF 1442 kb)

Table S1 (DOCX 38 kb)

## Data Availability

Data are available upon request.
