# Supplementary figures and images for: Thinner temporal peripapillary retinal nerve fibre layer in Stargardt disease detected by optical coherence tomography
Source: Graefes Arch Clin Exp Ophthalmol. 2020 Nov 13;259(6):1521–8. doi: 10.1007/s00417-020-04992-2 (PMC8166683; doi:10.1007/s00417-020-04992-2)

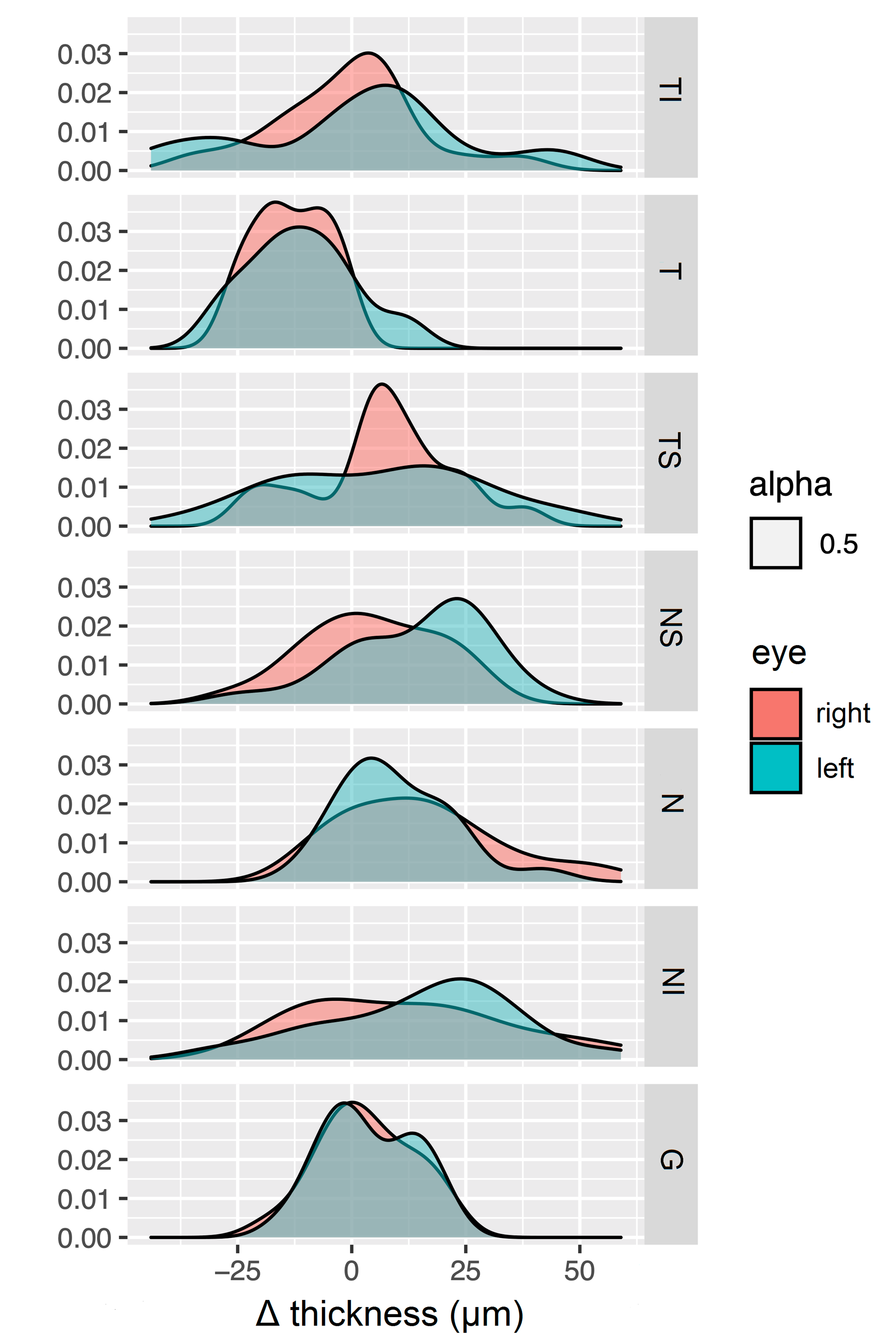

Supplement: Supplementary file 1 — Peripapillary RNFL thickness in Stargardt disease separated between the right and left eye. Differences in peripapillary RNFL thickness (Δ thickness) compared to age-corrected, normative data provided by the manufacturer (Heidelberg Engineering) of each sector are illustrated separately for the right and the left eye. (PNG 61788 kb) [file 417_2020_4992_Fig3_ESM.png]

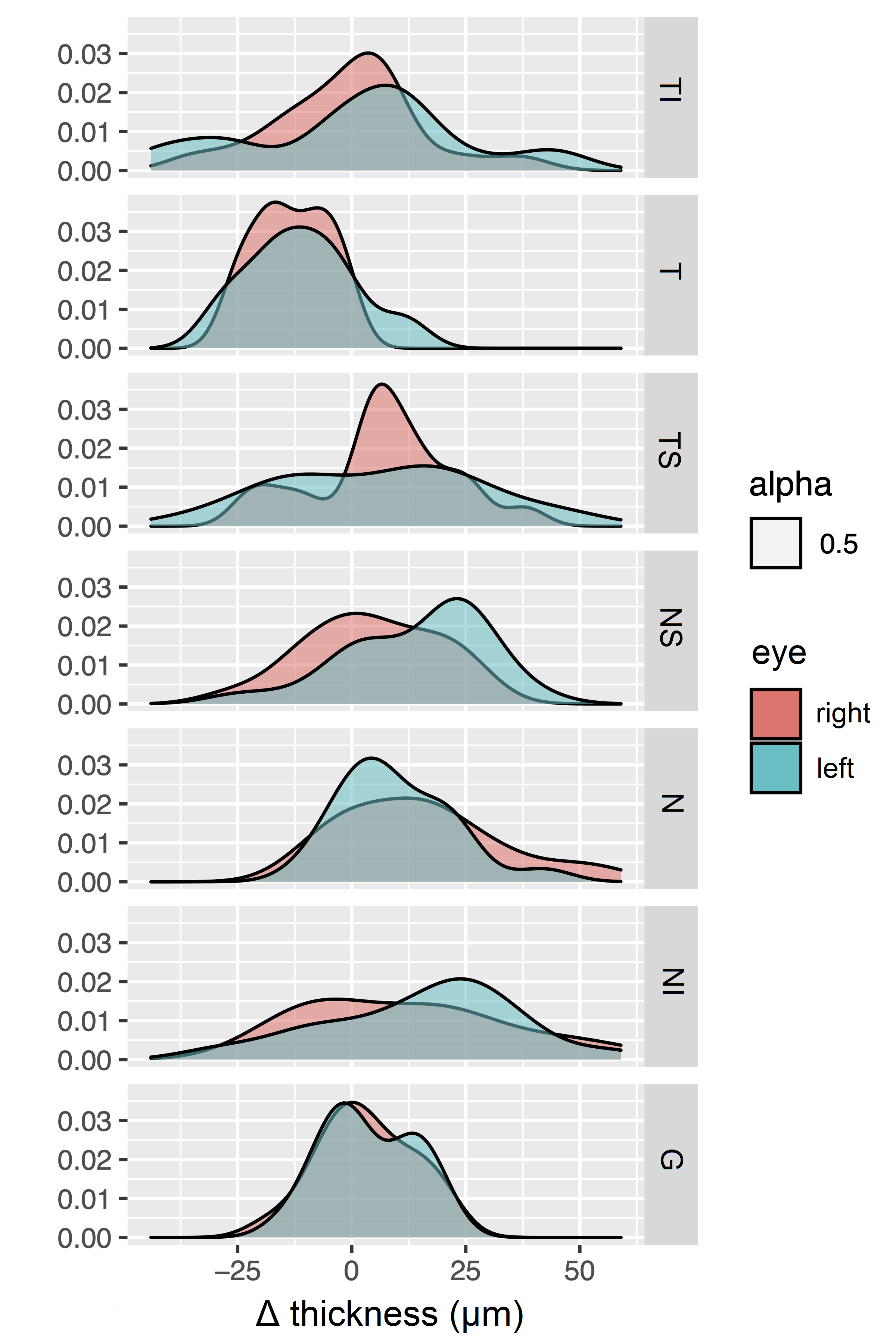

Supplement: Supplementary file 2 — High resolution image (TIFF 1287 kb) [file 417_2020_4992_MOESM1_ESM.tiff]

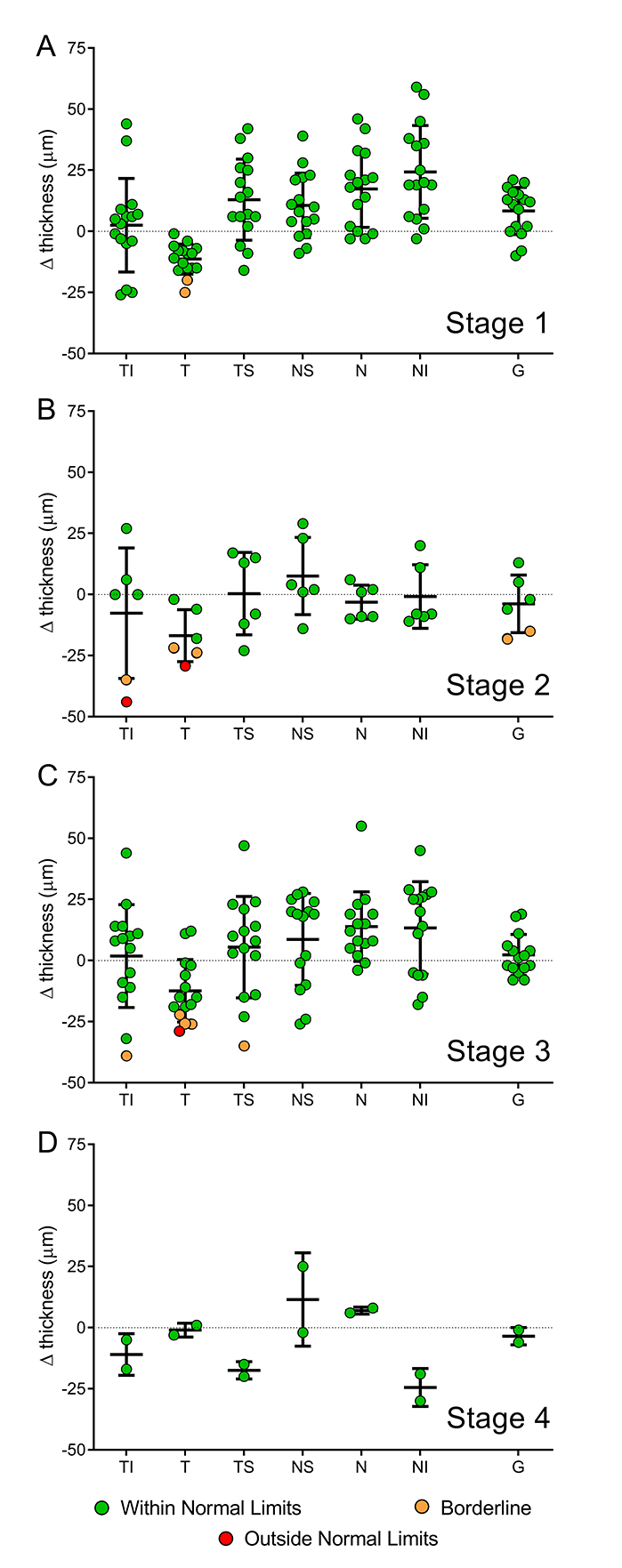

Supplement: Supplementary file 3 — Peripapillary RNFL thickness in different stages of Stargardt disease. The four STGD stages according to the Fishman’s STGD classification are shown (A-D). Differences in peripapillary RNFL thickness (Δ thickness) compared to age-corrected, normative data provided by the manufacturer (Heidelberg Engineering) of each sector are illustrated. “Within normal limits” (green plots) is defined as the area above the 5th percentile of the RNFL thickness distribution in eyes in the reference database, “borderline” (yellow plots) as the area below the 5th percentile, but above the 1st percentile and “outside normal limits” (red plots) as the area below the 1st percentile. Mean and standard deviation are shown. (PNG 3615 kb) [file 417_2020_4992_Fig4_ESM.png]

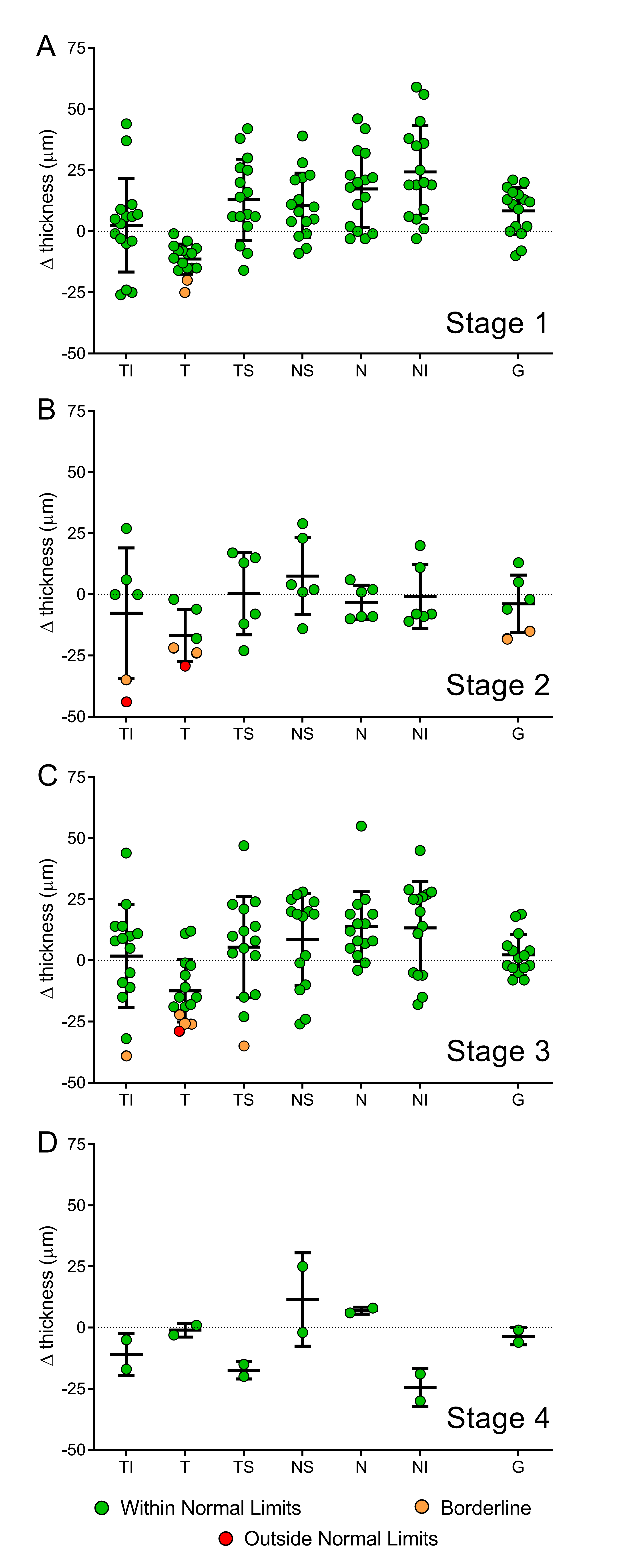

Supplement: Supplementary file 4 — High resolution image (TIF 1621 kb) [file 417_2020_4992_MOESM2_ESM.tif]

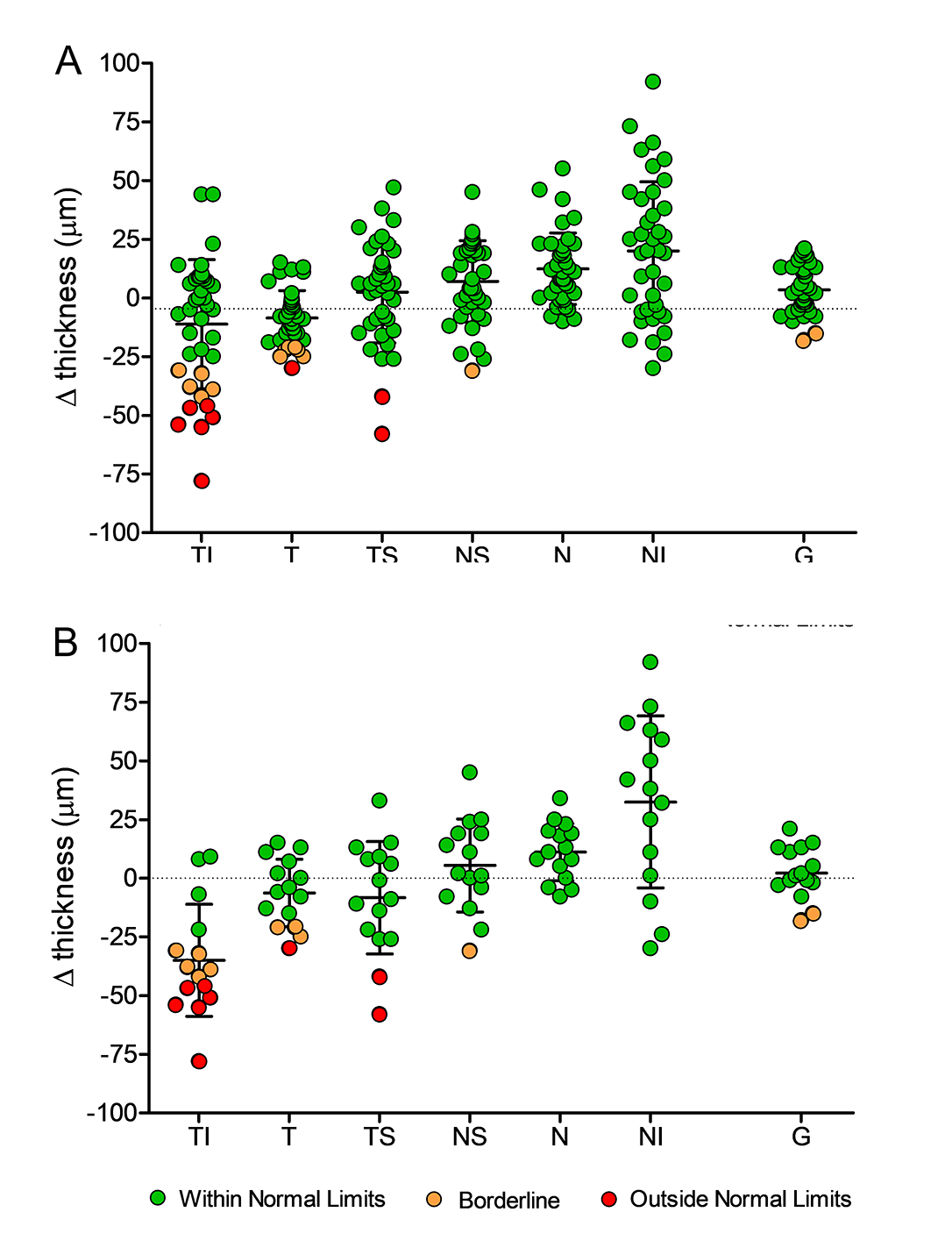

Supplement: Supplementary file 5 — Peripapillary RNFL thickness in different stages of Stargardt disease before correction of the optic disc-fovea angle. Data of all 39 eyes before correction of the optic dis-fovea angle (A) and only that 15 eye with misalignment of the optic dis-fovea angle (B) are illustrated. Differences in peripapillary RNFL thickness (Δ thickness) compared to age-corrected, normative data provided by the manufacturer (Heidelberg Engineering) of each sector are illustrated. “Within normal limits” (green plots) is defined as the area above the 5th percentile of the RNFL thickness distribution in eyes in the reference database, “borderline” (yellow plots) as the area below the 5th percentile, but above the 1st percentile and “outside normal limits” (red plots) as the area below the 1st percentile. Mean and standard deviation are shown. (PNG 4557 kb) [file 417_2020_4992_Fig5_ESM.png]

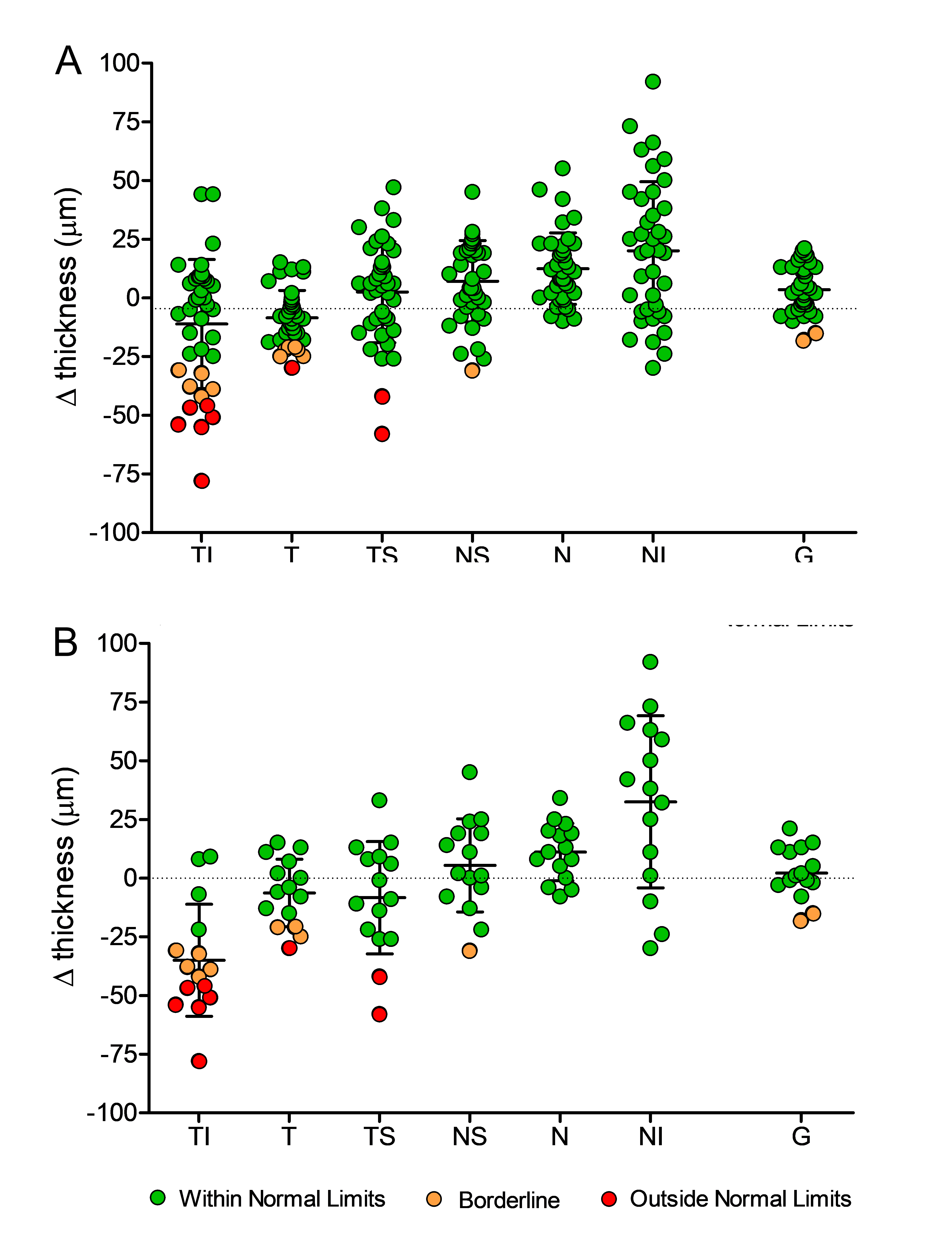

Supplement: Supplementary file 6 — High resolution image (TIF 1442 kb) [file 417_2020_4992_MOESM3_ESM.tif]
