# Supplementary material for: Thinner temporal peripapillary retinal nerve fibre layer in Stargardt disease detected by optical coherence tomography
Source: Graefes Arch Clin Exp Ophthalmol. 2020 Nov 13;259(6):1521–8. doi: 10.1007/s00417-020-04992-2 (PMC8166683; doi:10.1007/s00417-020-04992-2)
Supplement: Supplementary file 7 — (DOCX 38 kb) [file 417_2020_4992_MOESM4_ESM.docx]

**Table S1. Peripapillary thickness in different stages of Stargardt disease**

|  | **All Eyes**  **n=39**  **(µm, mean±SD)** | | **Stage 1**  **n=16**  **(µm, mean±SD)** | | **Stage 2**  **n=6**  **(µm, mean±SD)** | | **Stage 3**  **n=15**  **(µm, mean±SD)** | | **Stage 4**  **n=2**  **(µm, mean±SD)** | |
| --- | --- | --- | --- | --- | --- | --- | --- | --- | --- | --- |
|  | **thickness** | **∆ to control** | **thickness** | **∆ to control** | **thickness** | **∆ to control** | **thickness** | **∆ to control** | **thickness** | **∆ to control** |
| **TI** | 142±19 | 0±21 | 147±18 | 3±19 | 139±27 | -8±27 | 140±19 | 2±21 | 129±8 | -11±8 |
| **T** | 63±10 | -12±10 | 64±7 | -11±6 | 61±10 | -17±11 | 61±12 | -12±13 | 72±3 | -1±3 |
| **TS** | 142±19 | 7±19 | 149±16 | 13±17 | 138±17 | 0±17 | 139±21 | 5±21 | 116±4 | -18±4 |
| **NS** | 111±16 | 9±16 | 113±13 | 11±13 | 110±16 | 8±16 | 110±19 | 9±19 | 114±19 | 12±19 |
| **N** | 84±15 | 12±15 | 89±16 | 17±16 | 68±7 | -3±7 | 86±14 | 14±14 | 79±1 | 7±1 |
| **NI** | 119±22 | 14±21 | 131±19 | 24±19 | 110±12 | -1±13 | 116±21 | 13±19 | 81±7 | -25±8 |
| **G** | 101±10 | 3±10 | 106±9 | 8±10 | 95±11 | -4±12 | 100±10 | 2±8 | 93±4 | -4±4 |
